# Publisher Correction: Echo-Vision-FM: a pre-training and fine-tuning framework for echocardiogram video vision foundation model

**DOI:** 10.1038/s41467-026-68722-8

**Published:** 2026-01-20

**Authors:** Ziyang Zhang, Qinxin Wu, Sirui Ding, Xiaolong Wang, Jiancheng Ye

**Affiliations:** 1 Department of Electrical and Computer Engineering, Northwestern University, Evanston, IL USA; 2 Polytechnic Institute, Zhejiang University, Hangzhou, Zhejiang China; 3 Bakar Computational Health Sciences Institute, University of California San Francisco, San Francisco, CA USA; 4 Weill Cornell Medicine, Cornell University, New York, NY USA

**Keywords:** Health care, Translational research, Cardiology

Correction to: *Nature Communications* 10.1038/s41467-025-66340-4, published online 11 December 2025

In the version of the article initially published, there were errors in the Fig. 1c key. The labels “EchoNet-Dynamic | MAE:4.42(4.25-4.59)” and “CAMUS|MAE:3.87(3.86-3.88)” have now been corrected to “EchoNet-Dynamic | MAE:3.87(3.86-3.88)” and “CAMUS|MAE:4.42(4.25-4.59)” in the HTML and PDF versions of the article.

