# Supplementary material for: Echo-Vision-FM: a pre-training and fine-tuning framework for echocardiogram video vision foundation model
Source: Nat Commun. 2025 Dec 11;17:19. doi: 10.1038/s41467-025-66340-4 (PMC12764486; doi:10.1038/s41467-025-66340-4)
Supplement: Supplementary file 1 — Supplementary Information [file 41467_2025_66340_MOESM1_ESM.pdf]

Supplementary

1. Comparison of main results

The performance comparison for morphological value estimation (e.g, LV_{EF} , LV_{ESV} , LV_{EDV}) across all models used is visualized in Supplementary Figure 1. It is important to note that during training, LV_{ESV} and LV_{EDV} were logarithmically scaled for stability. However, when testing on the held-out set, the predicted values are rescaled back to their raw range to compute the evaluation metrics. Figure Supplementary 2 presents the ROC curves for heart function and disease diagnosis tasks, allowing a direct comparison of model performance. Supplementary Figure 3 demonstrates that Echo-Vision-FM continues to outperform other deep learning models across the same tasks, even when evaluated on different datasets.

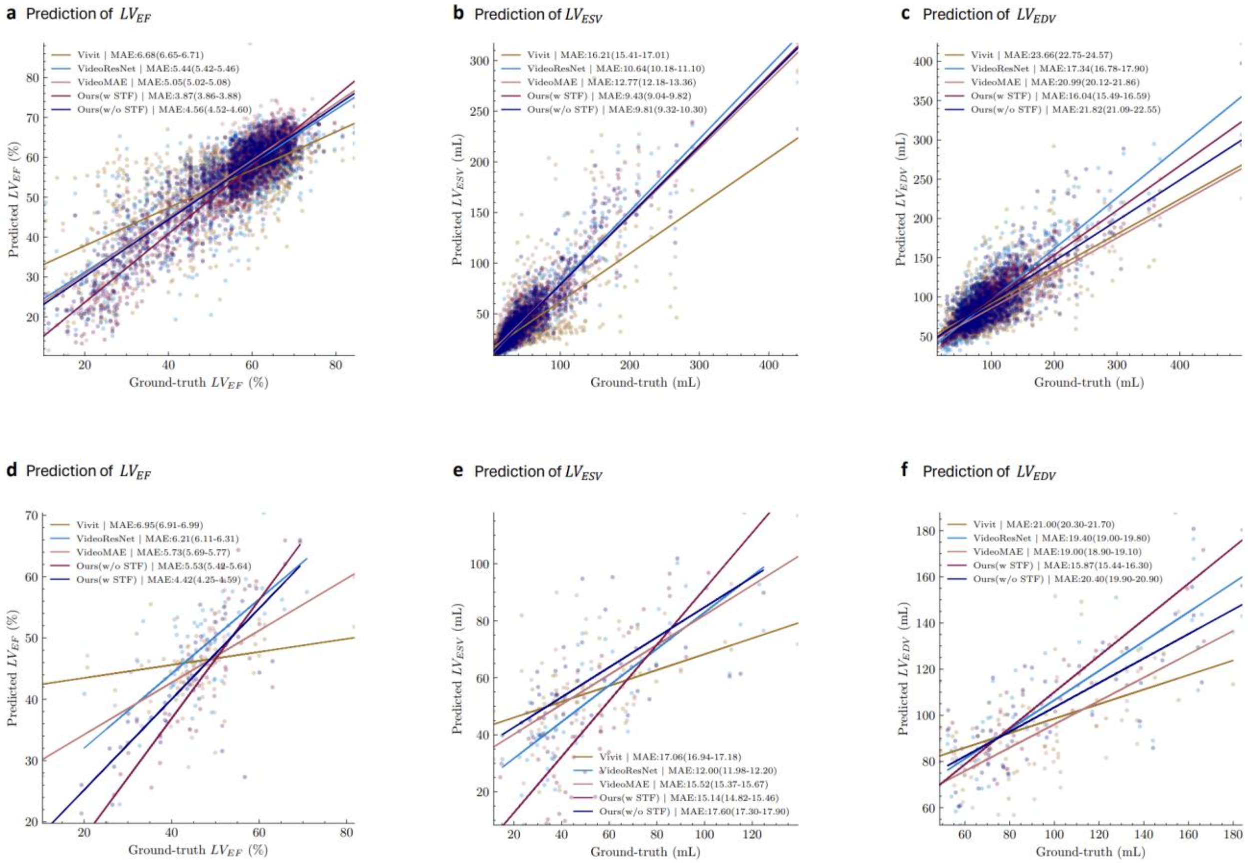

Figure 1. Comparison of all models on cardiac morphological value estimation. **a**, **b**, **c**: Scatter plots of predicted versus actual values for LV_{EF} , LV_{ESV} , and LV_{EDV} on the test set from Stanford Healthcare (EchoNet-Dynamic). **d**, **e**, **f**: Scatter plots of predicted versus actual values for LV_{EF} , LV_{ESV} , LV_{EDV} on the test set from the University Hospital of St. Etienne (CAMUS).

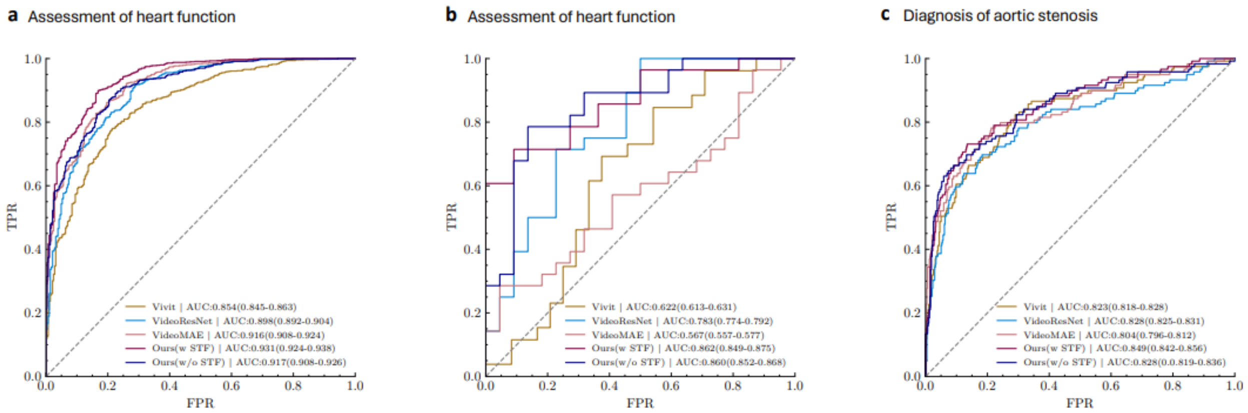

Figure 2. Comparison of all models on heart function and disease diagnosis. **a**, **b**: AUC performance for heart function diagnosis (classifying LV_{EF}) on held-out test dataset from Stanford Healthcare (EchoNet-dynamic), University Hospital of St. Etienne (CAMUS). **c**: AUC performance for diagnosis of aortic stenosis (e.g. "no_as", "mild_as", "mild-to-moderate_AS", "moderate_AS", "severe_AS") on the Tufts Medical Center (TMED) dataset.

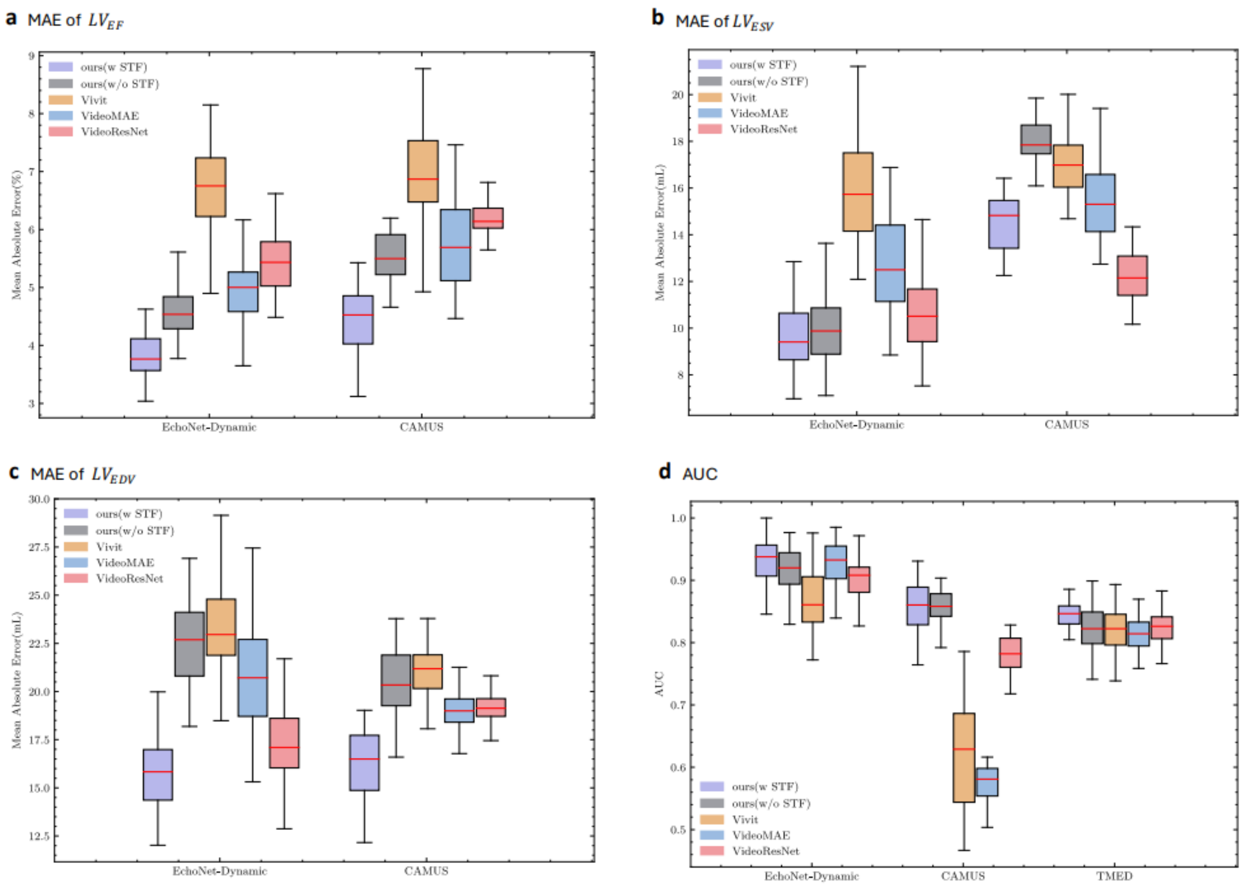

Figure 3. Box-plots of different clinical tasks. The center lines represent the median, and the boxes extend from the first to the third quartile.

2. Computational complexity

We summarized the computational complexity of both classification and regression models, with and without STF-Net, in Supplementary Table 1. All models achieved an inference speed of approximately 6 milliseconds per video, highlighting the practical efficiency and real-time applicability of our proposed approach.

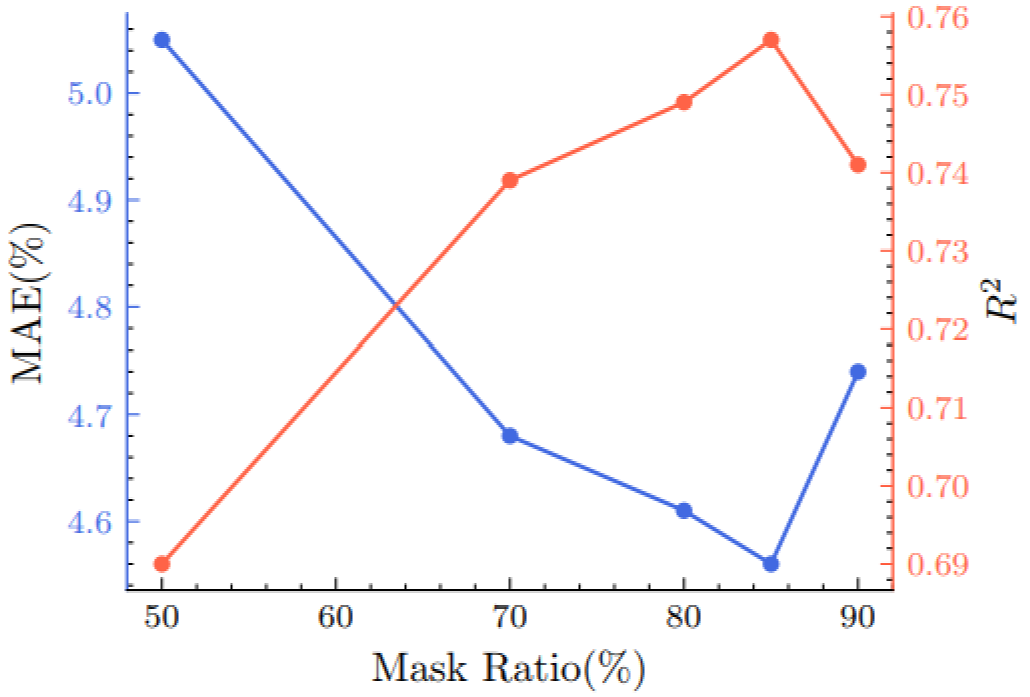

Figure 4. A double y-axis line chart illustrating the LV_{EF} regression results on the EchoNet-Dynamic held-out test set. The blue line represents the MAE, and the red line represents the r^2 values across different mask ratios. The chart demonstrates that the model achieved the best performance at a mask ratio of 85%.

Table 1. Computation complexity of proposed models. We report the number of floating-point operations (FLOPs), throughput (videos processed per second), and average inference latency per video (in milliseconds). All metrics are measured on a single NVIDIA H100 GPU.

	FLOPs	Throughput (videos/s)	Inference speed (ms/video)
Classifier (w/o STF-Net)	180.49G	169.06	5.92
Classifier (w STF-Net)	194.09G	151.39	6.61
Regressor (w/o STF-Net)	180.49G	169.73	5.89
Regressor (w STF-Net)	194.09G	153.69	6.51
